# Couch potatoes to jumping beans: A pilot study of the effect of active video games on physical activity in children

**DOI:** 10.1186/1479-5868-5-8

**Published:** 2008-02-07

**Authors:** Cliona Ni Mhurchu, Ralph Maddison, Yannan Jiang, Andrew Jull, Harry Prapavessis, Anthony Rodgers

**Affiliations:** 1 Clinical Trials Research Unit, University of Auckland, Auckland, New Zealand; 2 Department of Kinesiology, University of Western Ontario, London, Canada

## Abstract

The primary objective of this pilot study was to evaluate the effect of active video games on children's physical activity levels.

Twenty children (mean ± SD age = 12 ± 1.5 years; 40% female) were randomised to receive either an active video game upgrade package or to a control group (no intervention). Effects on physical activity over the 12-week intervention period were measured using objective (Actigraph accelerometer) and subjective (Physical Activity Questionnaire for Children [PAQ-C]) measures. An activity log was used to estimate time spent playing active and non-active video games.

Children in the intervention group spent less mean time over the total 12-week intervention period playing all video games compared to those in the control group (54 versus 98 minutes/day [difference = -44 minutes/day, 95% CI [-92, 2]], *p *= 0.06). Average time spent in all physical activities measured with an accelerometer was higher in the active video game intervention group compared to the control group (difference at 6 weeks = 194 counts/min, *p *= 0.04, and at 12 weeks = 48 counts/min, *p *= 0.06).

This preliminary study suggests that playing active video games on a regular basis may have positive effects on children's overall physical activity levels. Further research is needed to confirm if playing these games over a longer period of time could also have positive effects on children's body weight and body mass index.

ACTRN012606000018516

## Background

**Figure 1:**
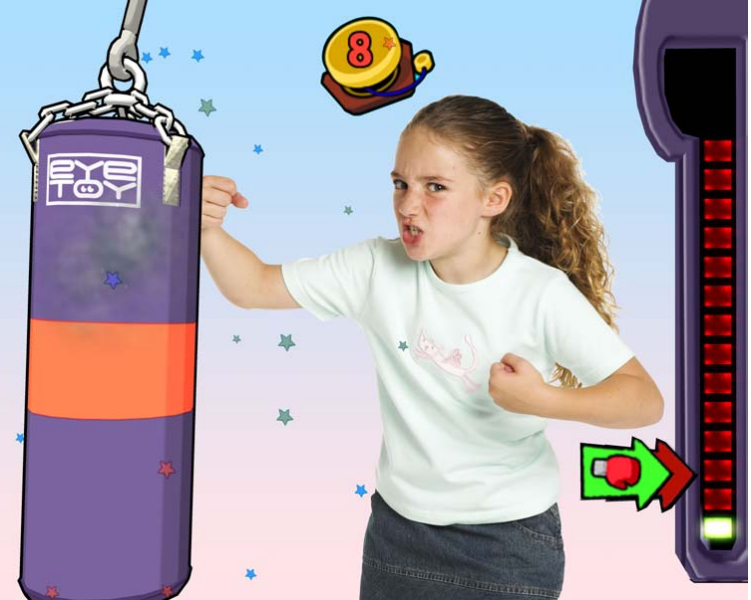
**Example of Child Playing EyeToy^® ^Knockout Active Video Game**. Reproduced with permission from Sony Computer Entertainment UK.

Widespread societal changes have increased time spent by children in screen-based activities, such as watching television, playing video games, and using computers[1]. There is now considerable evidence that watching television is associated with obesity in children and that other screen-based activities (such as computer use) may also have a similar association[2,3]. A new generation of active video games ('exer-games') such as Sony EyeToy^® ^and Dance Simulation products (Dance Dance Revolution™ [DDR] or Dance UK™) may, however, provide a novel strategy to increase physical activity levels in children. These games use a USB camera, which is placed on top of a television screen to place the players onscreen in the centre of the games (Figure 1). Players then physically interact with images onscreen in games that range from sport-based activities such as football and boxing, to dancing and kung fu. The game is dependent on player movement in front of the camera, for both control and actual gaming.

There is an emerging body of exer-gaming literature. Much of which, has evaluated the energy costs associated with dance simulation [4-6], EyeToy[7], arcade games[8], and use of various video games among those with spinal cord dysfunction[9,10]. Research has shown that energy expended by children playing active video games over short periods of time (2.3–5.0 child-specific METS)[7] is comparable to that expended in other moderate to vigorous physical activities such as brisk walking, skipping, jogging, and stair climbing[11,12]. Although computer games have been explored as an intervention for behaviour change [13-15], to the best of our knowledge only one previous study has examined the role of exer-gaming as potential intervention[16]. That study suggested DDR use was not related to change in BMI in overweight children and adolescents[16]. This may, however, have been related to the infrequent use of the DDR games. We are unaware of any studies that have examined the use of Sony EyeToy games as a possible intervention. Hence, this pilot study was conducted to evaluate the feasibility of a large randomised controlled trial (RCT) of the effects of active video games on body composition in children. The potential impact of such games on children's body composition is dependent on their effects on physical activity levels. Thus, specific research questions for the pilot included:

1. How often and for how long do children play active video games over a 12-week period?

2. What impact do active video games have on children's physical activity, measured both objectively and by self-report?

## Methods

A 12-week pilot randomised intervention study was conducted in Auckland, New Zealand, between April and July 2006. The 12-week intervention period was chosen because this was considered sufficient time to observe changes in physical activity and video game play and inform the feasibility of a large scale trial. The study protocol and related documents were approved by the Northern Regional Ethics Committee.

### Study participants and recruitment

Participants were recruited via community advertisements, direct contact with local schools, and word of mouth. Children were eligible for inclusion if they met the following criteria: aged between 10 and 14 years; owned a PlayStation^®^2 console; English speaking; and able to provide informed assent and parental consent. Children who already owned and played active video games, or who were unable to perform physical activity for medical reasons were excluded.

### Randomisation and interventions

Following informed consent and baseline data collection, participants were randomised to one of two groups: supply of an active video game upgrade package consisting of an EyeToy^® ^camera, EyeToy^® ^active games, and dance mat, or control (no intervention). Participants and their parents or guardians were instructed to substitute usual non active video game play with active video games (EyeToy^® ^and dance mat). The control group received an active game upgrade package upon completion of the study. Randomisation stratified by sex was carried out using a central web-based electronic randomisation service.

### Data collection

Participants were fitted with an Actigraph Accelerometer (Model AM7164-2.2C), which measures motion in the vertical plane, with movement outside of 'normal' motion being filtered electronically. Children wore the accelerometer during waking hours on their right hip at the mid-axilla line and activity count data (counts/minute) were collected over four consecutive days (two weekdays and two weekend days) at baseline (prior to randomisation), week six, and week 12. Minute-by-minute activity counts were uploaded to a data reduction program that excluded all Actigraph outputs that equalled zero for more than 20 consecutive minutes (assuming non-wearing time for that period). All days with less than eight hours of recorded time were excluded from analyses. Using these criteria, all participants provided at least three valid days (including one weekend day) for analysis. Time spent in light (1.5–2.9 METS), moderate (3.0–5.9 METS), and vigorous (≥ 6.0 METS) activities were derived from age-specific count cut-offs developed by Freedson et al[17].

Over the same four days, participants completed a daily activity record of estimated time spent playing electronic video games (active and inactive) as well as estimated time spent in various activities. Children were asked to record the type and time spent in physical activity (such as games, dance, sports etc) and video game play (active and nonactive) each day. MET values were assigned to each activity according to the Ainsworth compendium[18] and using cultural specific METs for indigenous activities[19].

Participants also completed the Physical Activity Questionnaire for Children (PAQ-C), a validated self-report seven-day recall physical activity measure, consisting of nine items that are used to calculate summary activity scores. Items assess physical activity performed at school (physical education, recess, lunchtime), right after school, and at home (organised and recreational)[20]. Each PAQ-C statement is scored on a five-point scale with higher scores indicating higher activity levels. In this study total PAQ-C scores were estimated at baseline and again at week 12. Previous research[20] has reported mean scores of 2.96 and 3.44 for young females and males, respectively. Height (Harpenden Stadiometer, Chasmors Ltd, London) weight (Salter scales), and waist circumference were measured according to standardised procedures[21] at baseline and week 12.

### Statistical analysis

Repeated measurement analyses were employed to test the intervention effect on physical activity as measured at baseline, 6 weeks and 12 weeks. Continuous physical activity measures were analysed using mixed models to account for missing values in the dataset. Activity counts were analysed using a generalised repeated linear model with Poisson regression. Regression analysis of covariance (ANCOVA) was conducted to test the effect of intervention on change in PAQ-C scores, PA records, BMI and waist circumference (WC) at week 12 (post-intervention) from baseline. All analyses adjusted for baseline measurements and sex (stratification factor), and were performed using SAS (Statistical Analysis Systems) version 9.1.3 and S-PLUS 6.1 for Windows. Adjusted means are presented in text and raw data are presented in the Table.

## Results

### Recruitment and participant characteristics

Twenty children were randomised: 10 received an active video game upgrade package and 10 received no intervention. Follow-up data were available for all study participants (100%). On average, study participants were 12 (SD 1.5) years of age and 40% were female (Table 1). Their mean baseline BMI was 19.7 (SD 3.6) kg/m^2 ^and they played an average of 80 (SD 72) minutes of electronic games per day. Children in the control group were older on average compared with the intervention group (13 years versus 11 years) and also spent more time playing electronic games (96 min/day versus 65 min/day). Since allocation to intervention group was random these differences are likely to have arisen due to the small number of children included in the pilot.

**Table 1:** Baseline characteristics of study participants

**Characteristic**	**Intervention Group (n = 10)**	**Control Group (n = 10)**
Age, yr		
Mean ± SD	11 ± 1	13 ± 1
Median [1^st ^quartile, 3^rd ^quartile]	10.5 [10, 12]	13 [12.25, 13.75]
Gender, n (%)		
Females	4 (40%)	4 (40%)
Use of all video games, minutes/day*		
Mean ± SD	65 ± 55	96 ± 88
Median [1^st ^quartile, 3^rd ^quartile]	48 [31, 66]	80 [30, 104]
Physical activity counts measured by accelerometer, counts/minute		
Mean ± SD	490 ± 188	490 ± 203
Median [1^st ^quartile, 3^rd ^quartile]	425 [400, 544]	418 [358, 585]
Time spent in light activity, minutes/day		
Mean ± SD	635 ± 90	652 ± 134
Median [1^st ^quartile, 3^rd ^quartile]	670 [594, 695]	650 [522, 779]
Time spent in moderate activity, minutes/day		
Mean ± SD	97 ± 48	69 ± 23
Median [1^st ^quartile, 3^rd ^quartile]	86 [59, 131]	70 [52, 81]
Time spent in vigorous activity, minutes/day		
Mean ± SD	7 ± 8	7 ± 7
Median [1^st ^quartile, 3^rd ^quartile]	6 [2, 9]	3.4 [3, 11]
Self-reported physical activity		
Time spent in moderate activity, minutes/day		
Mean ± SD	94 ± 56	64 ± 64
Median [1^st ^quartile, 3^rd ^quartile]	90 [65, 125]	44 [19, 89]
Time spent in vigorous activity, minutes/day		
Mean ± SD	80 ± 54	98 ± 87
Median [1^st ^quartile, 3^rd ^quartile]	72 [34, 118]	49 [34, 159]
PAQ-C score		
Mean ± SD	3.2 ± 0.5	2.7 ± 0.8
Median [1^st ^quartile, 3^rd ^quartile]	3.3 [3, 3.5]	2.65 [2.2, 3]
Waist circumference, cm		
Mean ± SD	73 ± 10	69 ± 11
Median [1^st ^quartile, 3^rd ^quartile]	74 [68, 80]	65 [64, 70]
BMI, kg/m^2^		
Mean ± SD	20.4 ± 3.6	19.0 ± 3.6
Median [1^st ^quartile, 3^rd ^quartile]	20.4 [17.9, 22.2]	18.3 [16.9, 20.3]
Overweight, n (%)^#^	1 (10%)	4 (40%)

* Participants completed a four-day record of time spent playing electronic video games (active and inactive)

^# ^Using cut-offs by Cole et al[22]

Note: Data presented in the table are raw data i.e. unadjusted

### Video game playing

Over the 12-week intervention period, children in the intervention group spent less total time playing all video games compared to those in the control group (54 versus 98 minutes/day [difference = -44 minutes/day, 95% CI [-92, 2]], *p *= 0.06). Children in the intervention group also tended to spend more time playing active video games compared to those in the control group (41 compared with 27 minutes/day [difference = 14 minutes/day, 95% CI [-15, 43], *p *= 0.3). The average time spent playing inactive games in the intervention group was significantly lower compared to the control group (47 versus 99 minutes/day [difference = -52 minutes/day, 95% CI [-101, -2]], *p *= 0.04).

### Physical activity levels

On average participants provided 12.1 hours (SD 1.4) of activity count data per day. Physical activity (counts per minute) measured with an accelerometer was higher in the active video game intervention group compared to the control group (mean difference at 6 weeks = 194 counts/min [95% C.I. 32, 310], *p *= 0.04, and at 12 weeks = 48 counts/min [95% C.I. -153, 187], *p *= 0.6). There were no significant differences in time spent in moderate and vigorous physical activities between the two groups as measured by accelerometer (*p *> 0.4), record (*p *> 0.3), or mean PAQ-C (*p *> 0.3) scores. When all activity time was combined (light, moderate, and vigorous), boys were more active than girls (*p *< 0.05).

### Waist circumference and BMI

Encouraging trends in the intervention group towards reductions in weight and waist circumference were observed. The mean difference in body weight between groups from baseline to 12 weeks was -0.13 kg (95% C.I. -1.97, 1.7), *p *= 0.9, while the mean difference in waist circumference between groups from baseline to 12 weeks was -1.4 cm (95% C.I. -2.68, -0.04), *p *= 0.04. However, the pilot study was not adequately powered to detect differences in anthropometric outcomes.

## Conclusion

Results from this 12-week pilot study suggest that children randomised to an active video games upgrade undertake more physical activity, play fewer video games overall, and have decreased waist circumferences compared to controls. These findings suggest that, at least in the short-term, active video games may be an effective means to increase children's overall physical activity levels. Further research is needed to confirm if these games could also have a positive effect on children's body weight and BMI over a sustained period of time.

## Competing interests

The author(s) declare that they have no competing interests.

## Authors' contributions

CNM conceived of the study, oversaw its design and coordination, assisted in interpretation of the analyses, and drafted the manuscript. RM coordinated the study, undertook data collection, assisted in interpretation of the analyses, and helped to draft the manuscript. YJ participated in the design of the study and performed the statistical analyses. AJ, HP and AR participated in the design of the study, assisted in interpretation of the analyses and provided comment on the manuscript. All authors read and approved the final manuscript.
